# Accuracy of full-guided versus half-guided implant procedures carried out with digital implant planning software by students as part of a university curriculum

**DOI:** 10.1186/s12909-024-06280-7

**Published:** 2024-11-15

**Authors:** Daniel Einsiedel, Stephanie Knapp Giacaman, Anna Seidel, Lara Berger, Mayte Buchbender, Manfred Wichmann, Ragai Edward Matta

**Affiliations:** 1 Present Address: Department of Prosthodontics, University Hospital Erlangen of Friedrich-Alexander University Erlangen-Nürnberg (FAU), Glueckstrasse 11, 91054 Erlangen, Germany; 2 Department of Oral and Maxillofacial Surgery, University Hospital Erlangen of Friedrich-Alexander University Erlangen-Nürnberg (FAU), Glueckstrasse 11, 91054 Erlangen, Germany

**Keywords:** Dental education, Teaching methods, Dental implantation, Digital implant planning, Surveys and questionnaires, Students, Digital dentistry

## Abstract

**Background:**

This in vitro study investigated whether full-guided (FG) or half-guided (HG) implant placement is more suitable for beginners and to what extent the use of the coDiagnostiX (CDX) (10.5, Straumann Group, Basel, Switzerland) implant planning software proves useful in teaching.

**Methods:**

Twenty students planned implant positions with CDX which were then placed in a model using printed drill templates in the sense of FG implantation (group 1) and HG implantation (group 2). The implant positions could be compared with those of the reference model, and deviations could be determined. The results were tested for significance using the t-test for independent samples for groups 1 and 2. A total of 32 students subsequently completed a questionnaire about the software. Cronbach's α was also calculated to check the reliability of the questions for the individual subject areas.

**Results:**

In both groups the greatest deviation occurred along the y-axis in the vestibular direction. It measured 1.390 mm in group 1 and 1.570 mm in group 2. Comparing both groups, there were significant deviations along the y-axis (*p* = .013), along the z-axis (*p* = .049), and in the total deviation (*p* = .031). The questionnaire was evaluated in design, with 95% positive answers. In contrast, the evaluation of the area of time and effort resulted in only 55% positive responses. Overall, experience with the software was rated as positive by 74%.

**Conclusions:**

Group 1 achieved more accurate results, especially along the y-axis in the vestibular direction. In both groups, the implants were placed too deep. The questionnaire indicated a software with high usability and is therefore very suitable for teaching. If clinically feasible, beginners should prefer full-guided implant placement.

**Supplementary Information:**

The online version contains supplementary material available at 10.1186/s12909-024-06280-7.

## Background

Implant dentistry is becoming increasingly important both in modern dentistry and in university education [1–3]. The expectations of patients regarding their prosthetic care are also increasing. They hope that implant placement will result in a dental situation that is comparable to the original dentition [4]. For example, when a removable prosthesis is changed to single-tooth implantation in the esthetic area, a strong improvement in patient satisfaction is observed [5].

To provide a definitive restoration for a patient, conventional implant planning requires several steps, such as taking impressions or fabricating plaster models and special scanning prostheses [6]. Conventional two-dimensional imaging also cannot be used to determine the oro-vestibular width of the bone [7].

In contrast, digital implant planning involves three-dimensional (3D) imaging of the hard tissue, usually via cone beam computed tomography (CBCT). In addition, the intraoral situation including soft tissue is recorded using an intraoral scanner. These data can now be imported into implant planning software [8]. Depending on the provider, planning programs are either closed or open systems [9].

The software is then used to analyze the hard and soft tissues and to determine a suitable implant position. During planning, the software indicates, for example, undercutting of minimum distances from the previously marked nerve course [6]. The position can then be precisely transferred to the oral situation with the aid of a surgical template [8, 10]. Computer-guided implantation is superior to conventional implantation in terms of accuracy, morbidity, outcome, and efficiency [11, 12]. Regarding pedagogical methodologies in the university setting, students show a strong affinity and easy understanding of emerging technologies, exhibiting a rapid acquisition of knowledge about their application [13]. Consequently, it is imperative to integrate these aspects into modern education curricula.

A disadvantage of guided implantation is the limited oral space available with a restricted mouth opening, which may make it difficult or impossible to insert the surgical guide with the drill and handpiece simultaneously [11]. Furthermore, in a CBCT-based workflow, patient movement during the scan may result in inappropriate imaging [8]. Shorter scan protocols, with lower resolution are usually sufficient for implant planning, thus reducing the radiation dose and motion artifacts; they can be considered a solution for inappropriate imaging, especially if the scan needs to be repeated [14].

The practice team and the treating dentist should of course be qualified for a digital workflow in terms of software, hardware and practical knowledge [12]. Although current software guides the user through the planning process step-by-step and offers orientation and assistance at several points, the software does not counterbalance a lack of experience. It is important to compare the planning with the clinical situation and to critically question whether it is actually error-free and feasible in order to be able to modify it according to the situation if necessary. The responsibility remains in the hands of the practitioner [12, 14].

Guided surgery can assist in the exact realization of the planned position of the implant. It is divided into FG, HG, and freehand-guided (FH) surgery depending on the extent of template-guided implantation. FG surgery covers the complete implantation process. Thus, both the drilling and the insertion of the implant are determined by the surgical guide. In HG surgery, either the pilot drilling or the entire drilling sequence is performed with the drilling template. The drill guide is not used for the subsequent insertion of the implant. In contrast, no surgical guide is used for FH implantation. Although preoperative 3D planning is recommended, FH implantation is also possible without 3D radiography [11].

FG surgery is significantly more precise than HG and FH surgery are, with a smaller difference between FG and HG surgery [15, 16]. FG-flapless-surgery with a tooth-supported template is the most accurate method [11].

Digitalization consequently opens up a multitude of practical possibilities, but also creates new problems and areas of competence that must be acquired by practicing for both dentists and students. Therefore, an important part of the teaching of digital dentistry at university level is the early contact with the corresponding software and hardware, so that the students gain basic knowledge for later clinical routines and are able to expand it. Third-year students have already been surveyed on the use of implant software in their studies. As a result, a high acceptance of the software was found [17].

The aim of this in vitro study of the curriculum "Digital Dentistry" is to investigate which type of implant placement is most suitable for beginners and to what extent the CDX planning software is suitable for use in teaching. The null hypothesis for the study is that there is no significant difference in implant position between group 1 and 2.

## Methods

### Structure of the curriculum "Digital Dentistry"

This study was conducted in the Department of Prosthodontics at the dental school affiliated with Friedrich-Alexander University Erlangen-Nürnberg in Erlangen, Germany. All participants in the trial were undergraduate students in their 8th and 9th semesters of the 10-semester program, with no prior knowledge or experience in preoperative planning or implantation procedures. The offer to participate in the study was made to 35 students on the voluntary "Digital Dentistry" curriculum, 20 of whom decided to take part voluntarily and agreed to perform additional practical exercises for the study. The study participants had no advantage or benefit over the non-participants and vice versa. In addition, a questionnaire was completed by 32 students.

**Fig. 1:**
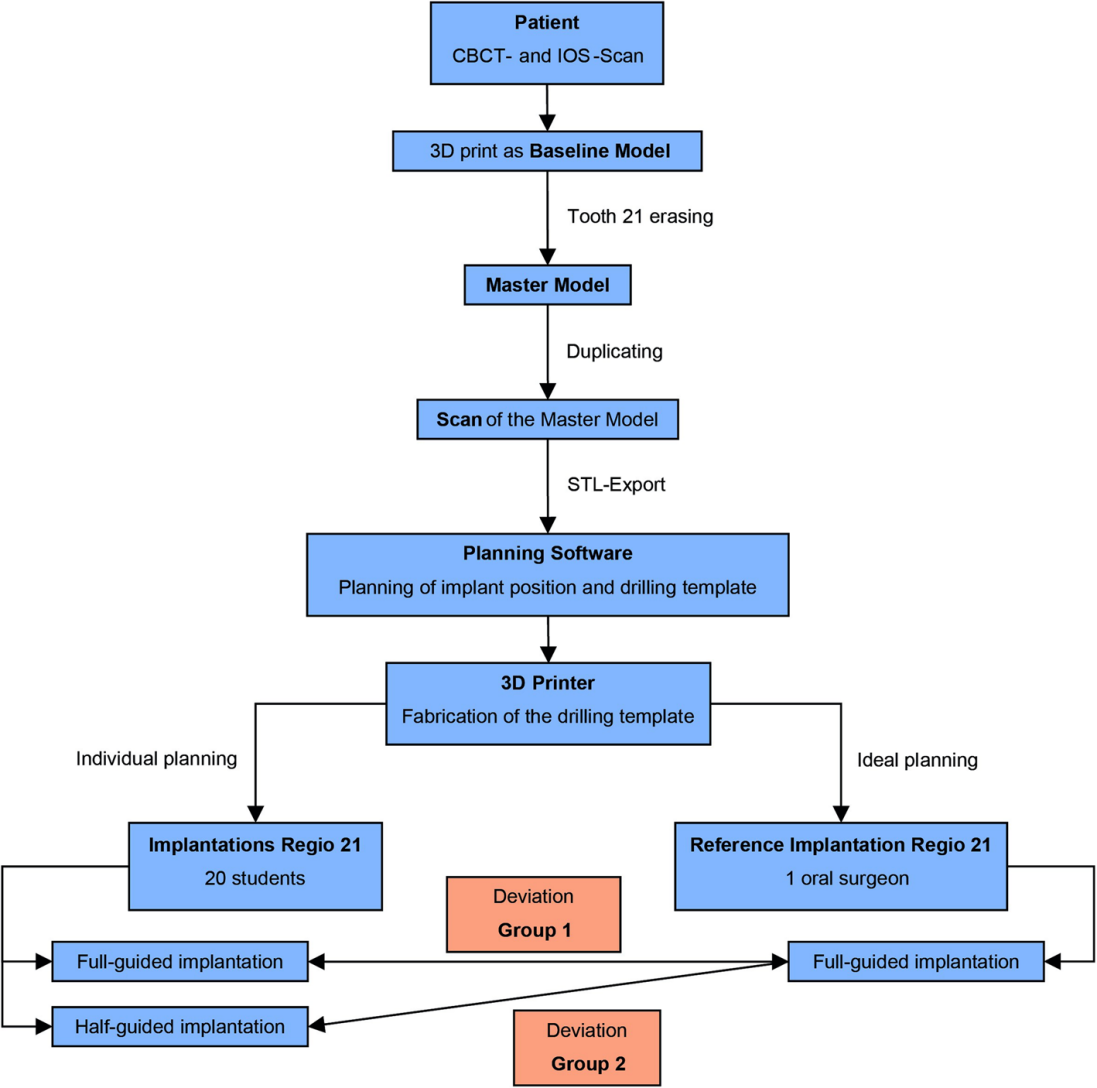
Flowchart for the study procedure and the comparison of groups 1 and 2

With the assistance of an experienced oral surgeon, the students were first instructed in detail in the use of the CDX software and the materials provided, such as the drill cassette and the surgical motor. The implant planning software was used in the "producer" license; therefore, it can be considered an open system. In the second step, a CBCT scan and an intraoral scan (IOS) were created with the help of a real patient from the dental clinic. The data were used to print the baseline model for the maxilla and mandible. These are fully toothed jaws without wisdom teeth. The tooth 21 was now erased in order to create a challenging situation in the esthetic area for the curriculum, where the positioning of the implant plays a decisive role. This master model could now be duplicated. For this purpose, the resin Biresin G27 (Sika Deutschland GmbH, Stuttgart, Germany) was mixed in a 1:1 ratio of "Komp. A" and "Komp. B beige". In addition, 7 g of microballoons (Modellbauzentrum, Erlangen, Germany) were added per 100 g of resin. The mixture was finally cured for 10 min in a pressure pot under 1 bar (Fig. 1).

**Fig. 2:**
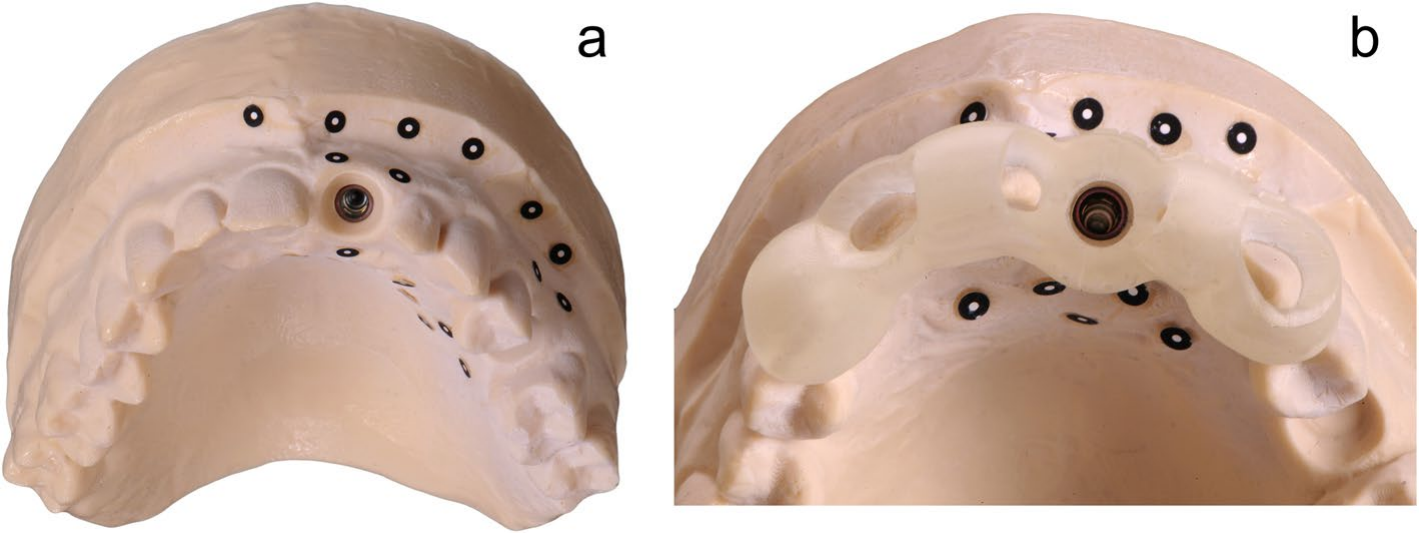
Model with **a**) inserted implant and **b**) surgical guide prepared for digitalization

**Fig. 3:**
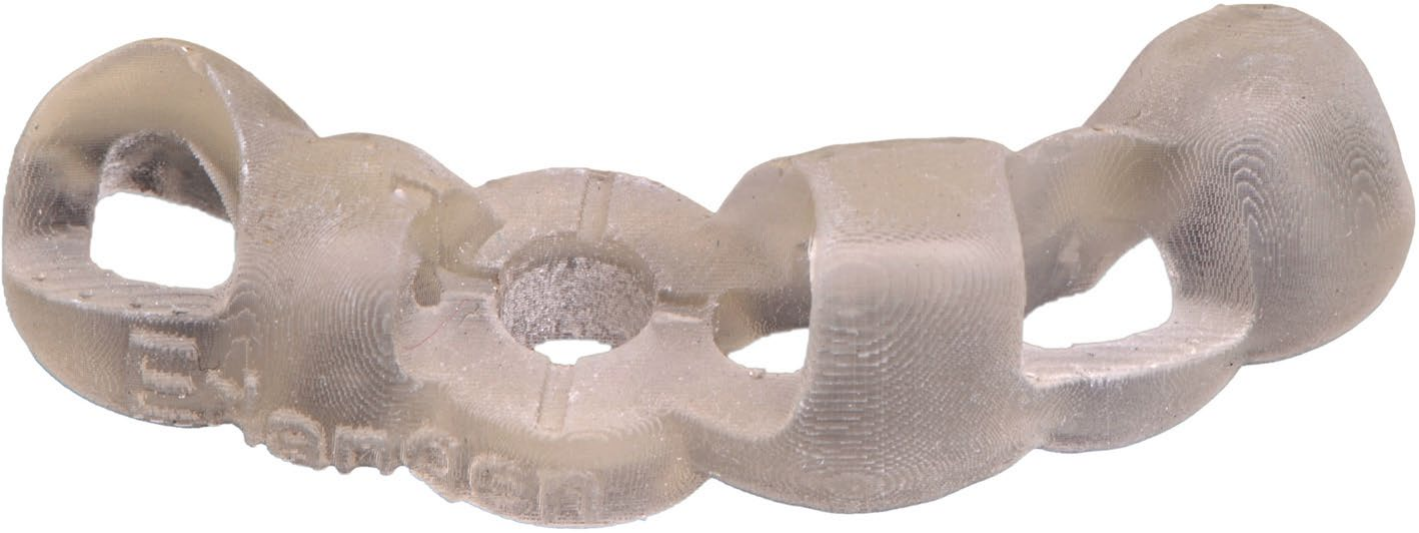
3D printed surgical guide based on the individual presurgical planning

In the curriculum, each student made a scan of their models using the Primescan AC (Dentsply Sirona, Charlotte, USA) and exported them as an STL file. This, in combination with the CBCT dataset of the patient in region 21, allowed each student to individually plan an implant placement with an associated surgical guide using the CDX software. Expert support was available to the students if needed. The designed surgical guides were then printed using the Asiga MAX UV (Asiga, Sydney, Australia). The resin used was Imprimo LC MJF (Scheu Group, Iser-lohn, Germany) with a layer thickness of 0.1 mm. The remaining parameters were deposited accordingly on the Asiga MAX UV (Asiga) for the material and were not modified further. With the appropriate drilling protocol, the Bone Level RC implant with a length of 10 mm and a diameter of 4.1 mm (Straumann Group, Basel, Switzerland) could be implanted in the sense of an FG surgery. Similarly, a second identical model was used to implant in region 21 according to the principles of HG surgery. The students were asked to mark the implant position on the alveolar ridge with a round bur using the previous surgical guide, remove the guide and then complete the implantation without further use of the drilling template (Figs. 2 and 3).

To obtain a reference dataset, the implantation conducted by an experienced oral surgeon was utilized. Initially, she determined the optimal implant position in region 21 using the preoperative planning data in the CDX software, which were the same data used by all the other study participants. Subsequently, a surgical guide for full-guided static implantation was virtually designed in accordance with the implant manufacturer's recommendations and produced with the 3D printer using the recommended resin. The template was then employed for implant placement in region 21 in the simulated patient model used by all study participants. This model with the corresponding implant position served as the reference for evaluating the students' implant positions.

### Digitalization and evaluation of the models

**Fig. 4:**
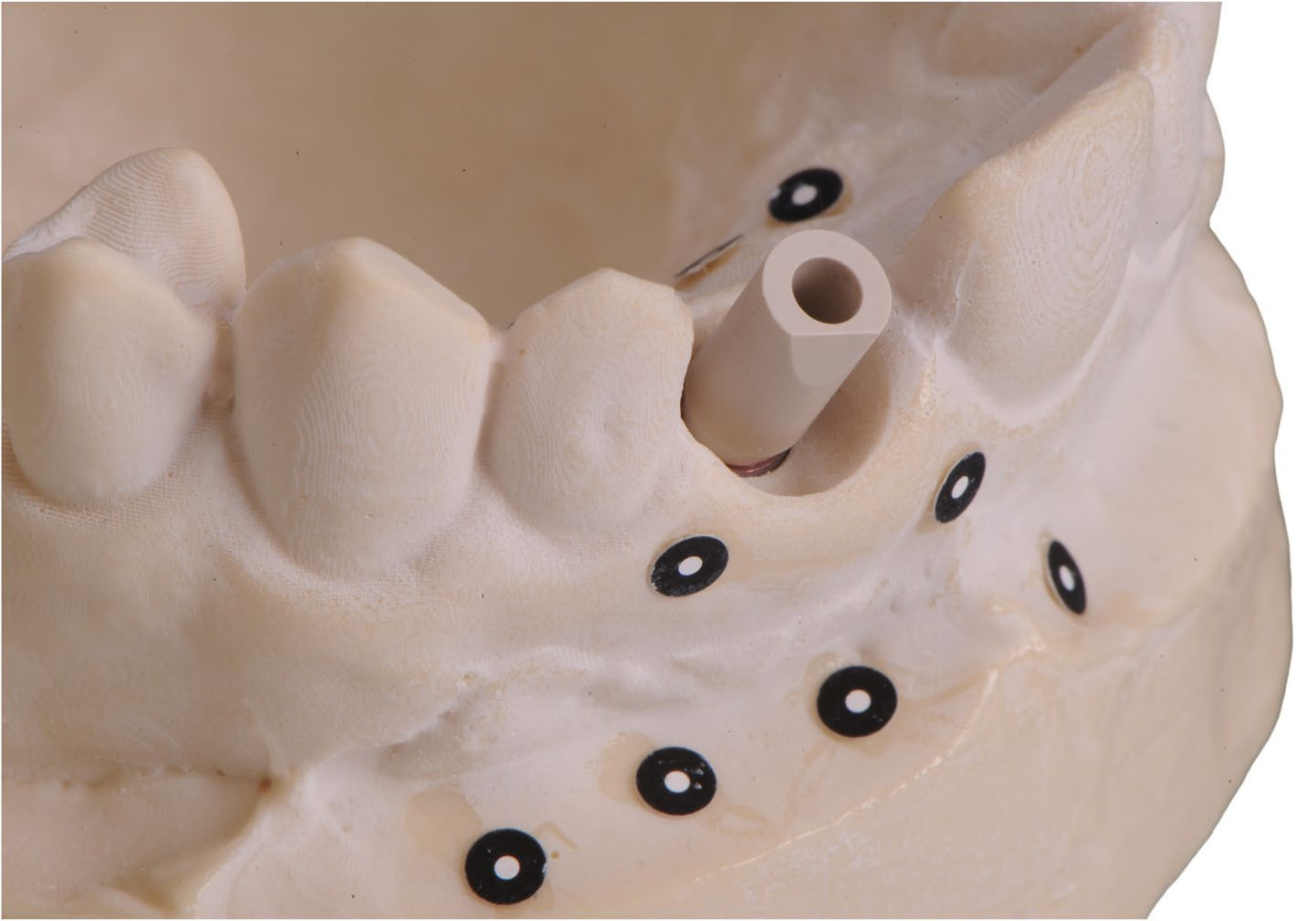
Model with fitted scanbody

The models were then fitted with the compatible Cares RC Mono Scanbody (Straumann Group, Basel, Switzerland). Using the ATOS III Triple Scan (GOM Metrology, Braunschweig, Germany) industrial scanner and the ATOS Professional (2018, GOM Metrology) software, the models were acquired at a constant room temperature of 20 °C and saved as STL (Fig. 4).

For the evaluation, the models can be divided into two groups. Group 1 includes a comparison of the students' FG implantation with the previously mentioned FG reference implantation for region 21. Group 2 also compares the HG implantation with the same FG reference implantation.

In the Gom Inspect Professional (2018, GOM Metrology) software, a coordinate system could now be inserted in the FG reference according to the principle "By 3–2-1". In addition, the "Fitting Cylinder" and "Fitting Plane" functions were used to provide the scanbody with a fitting cylinder and a plane marking the occlusal end. An intersection point could now be constructed between the two geometric figures, which was referred to as a reference for further measurements. This dataset was saved as a separate file in the "atos" format and used as a reference for the evaluation of all the models.

For the models of the two groups, the reference file was now opened with the Gom Inspect Professional (GOM Metrology) software and the model that was to be analyzed was imported. Matching with the reference was first performed via the "Prealignment" function. After selecting gap-bounding neighboring teeth, two mesial and two distal in each case, the "Local Best-Fit" function was subsequently used to achieve the most precise possible superimposition of the models on each other in the area to be examined.

As already described for the reference model, the scanbody of the model that was to be examined was also provided with a cylinder and a plane and an intersection point was constructed. Thus, the two intersection points could be compared with each other and the position deviation along the x, y and z axes and the total deviation XYZ could be determined (Fig. 1).

The deviations of the two groups could now be transferred to the statistical program IBM SPSS Statistics (28.0.1.1, IBM Corporation, Armonk, USA) and evaluated. The variances of the three axes were tested for significance using the t-test for independent samples for groups 1 and 2. In addition, the mean and standard deviation, as well as the maximum and minimum values were calculated.

### Structure and evaluation of the questionnaire

Following implant planning and execution, 32 of the students completed a questionnaire about the implantation software. It contained 26 questions, which were divided into the topics of design, assistance by the software, time and effort, ease of use, planning outcome and future use. The survey was created by the working group for this study, with the purpose of accurately reflecting the students' perceptions. Considering the novelty of the digital dentistry curriculum and the teaching method, the questions were tailored to align with the current curriculum and were based on the experiences gained from previous courses. The Likert scale was chosen as the survey instrument to respond to the questions, which ranged from a value of 1 (indicating “does not apply” or "strongly disagree") to 10 (indicating “applies” or "strongly agree"), with increments of 1. In order to be able to compare the answers, the scale was recoded for four questions, marked with “(†)” in Table 2. Scores from 1–3 were rated as "negative", those from 4–7 as "neutral", and those from 8–10 as "positive".

The questionnaires were collected at the end of the curriculum, transferred to a digital data spreadsheet (Microsoft Excel 2021, Microsoft Corporation) and analysed in a statistics programme (IBM SPSS Statistics 28.0.1.1, IBM Corporation). Cronbach's α was also calculated. Thus, the results of the individual questions as well as those of the respective areas could be investigated.

## Results

### Results of the model evaluation

**Fig. 5:**
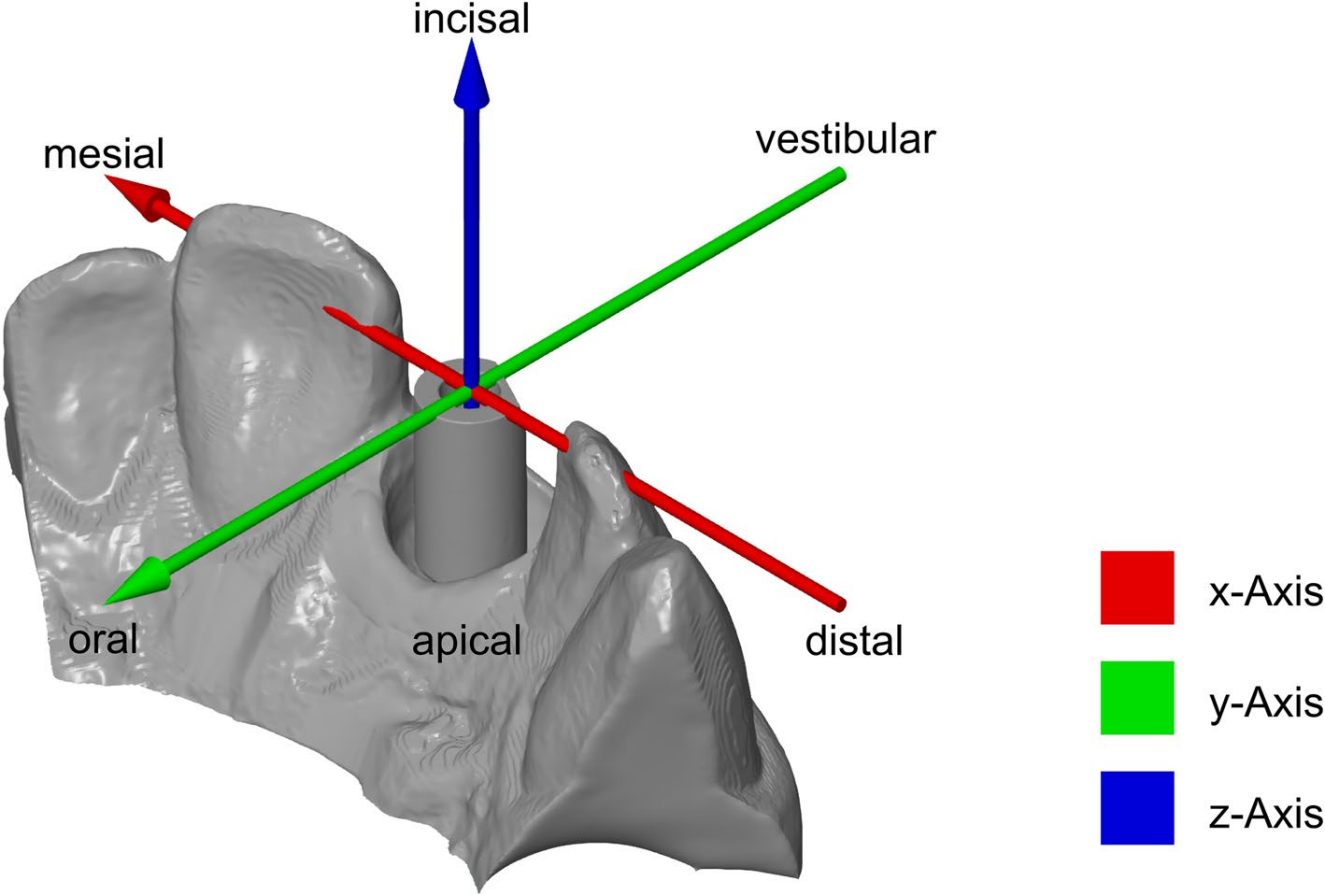
Visualization of the axes used in the evaluation

**Fig. 6:**
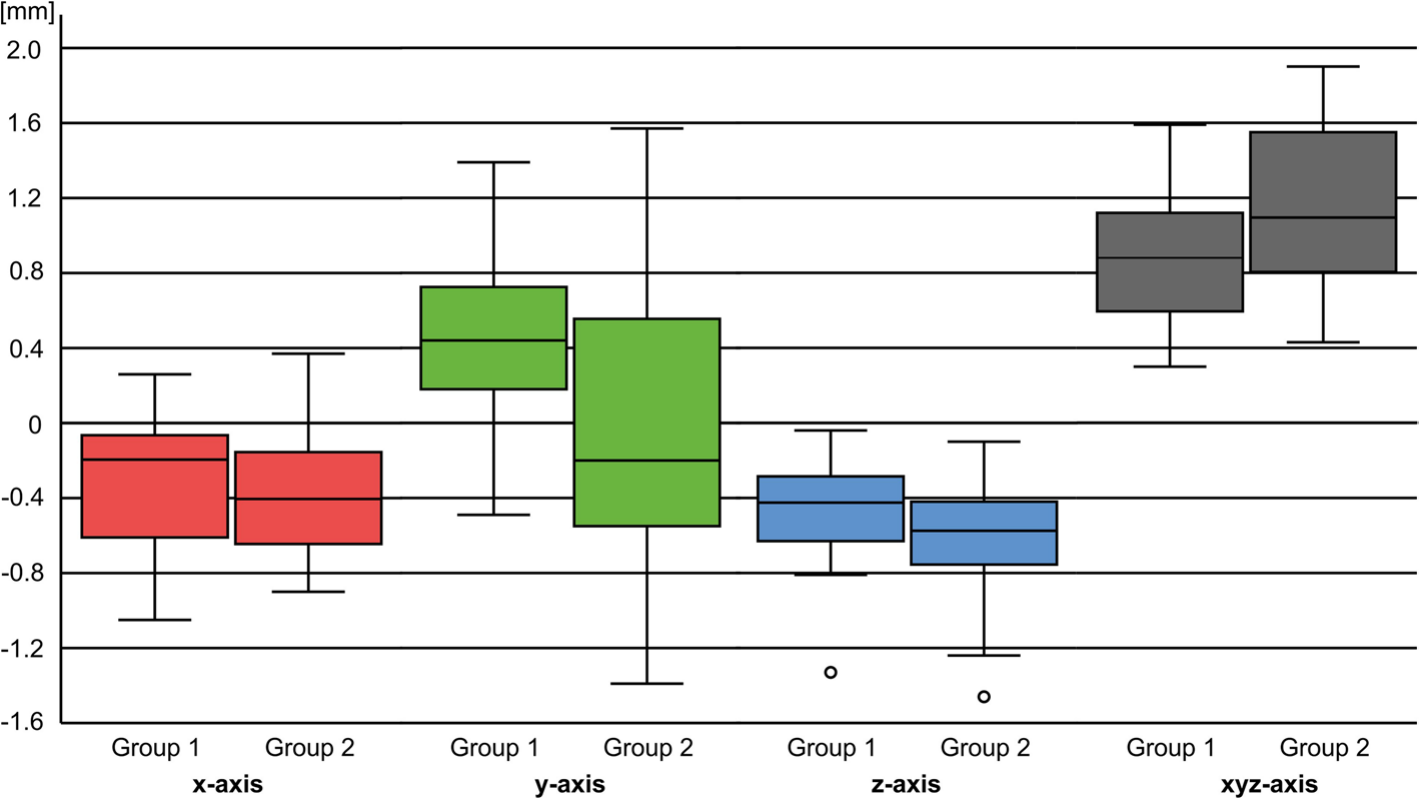
Boxplots of the deviations along the x/y/z/xyz axes

The x-axis describes deviations in the mesial ( +) or distal (-) direction, the y-axis in the oral ( +) or vestibular (-) direction, and the z-axis in the incisal ( +) or apical (-) direction (Fig. 5). The total deviation is described with XYZ (Fig. 6).

In group 1, the highest deviation from the zero line was 1.390 mm along the y-axis. The lowest deviation from the zero line was -0.040 mm along the z-axis. The total deviation was at least 0.300 mm and at most 1.590 mm. The largest standard deviation resulted from the y-axis values of 0.453 mm. The highest deviation from the zero line in group 2 was also along the y-axis at 1.570 mm. The lowest deviation from the zero line was along the z-axis at -0.100 mm. The total deviation was at least 0.430 mm and at most 1.900 mm. The largest standard deviation was also along the y-axis at 0.879 mm. The mean total deviation was 0.893 mm in group 1 and 1.146 mm in group 2 (Table 1).

**Table 1:**
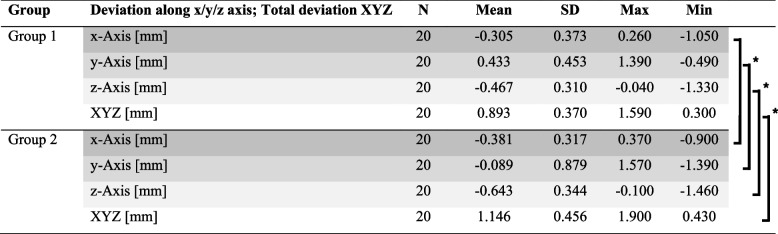
Deviations, means, standard deviations, maxima, and minima along the x, y, z and xyz axes in groups 1, 2, and 3; * = significant

Comparing group 1 and 2, the difference in the mean deviation was the absolute value of 0.076 mm along the x-axis, 0.344 mm along the y-axis, and 0.176 mm along the z-axis. The difference in total deviation was 0.253 mm.

Significant deviations were found along the y-axis (*p* = 0.013), along the z-axis (*p* = 0.049) and in the total deviation (*p* = 0.031) when comparing group 1 and 2. No significant deviation was detected along the x-axis.

**Fig. 7:**
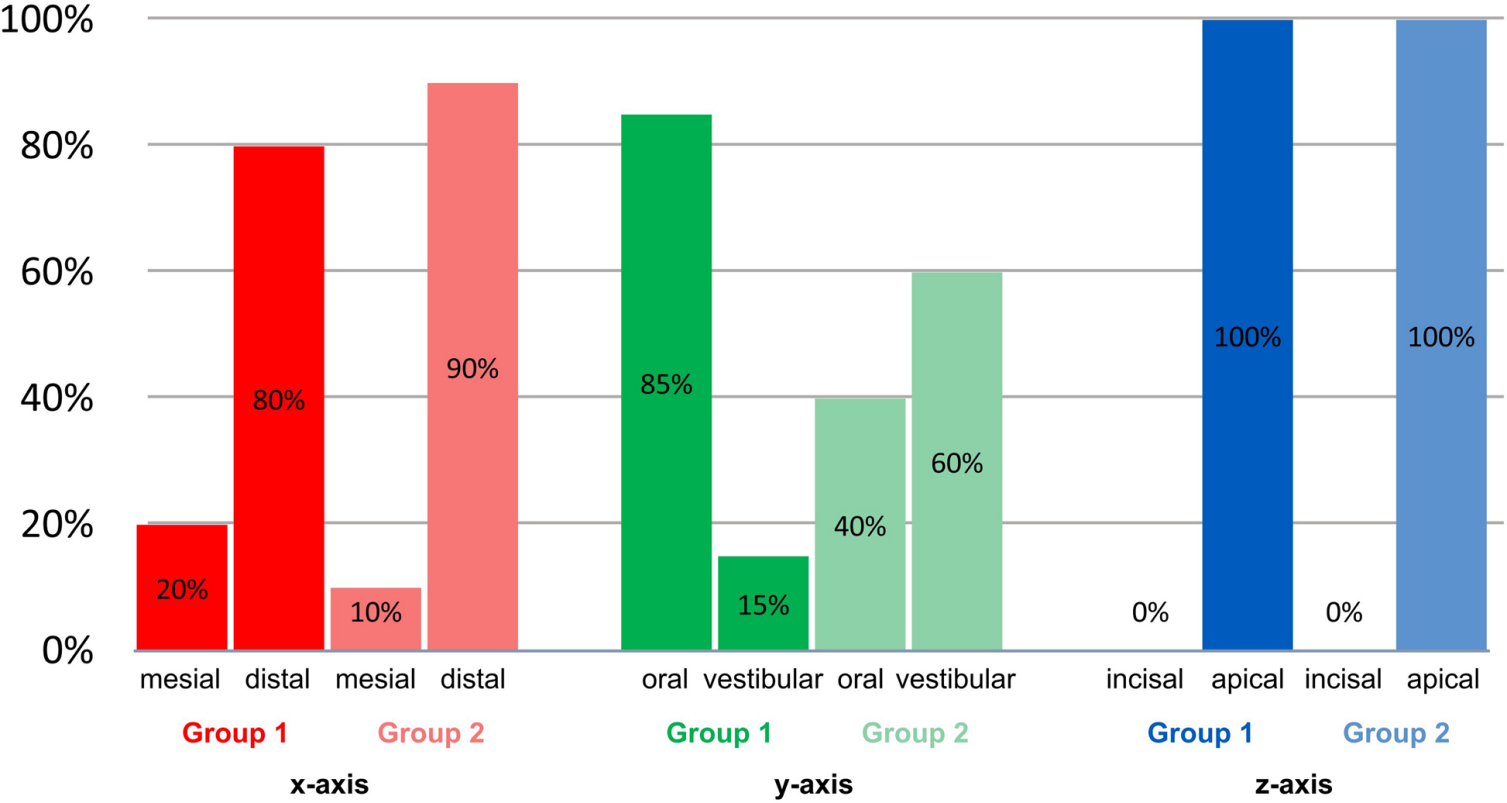
Percentage of the direction of deviation along the x/y/z axes

In both group 1 and group 2, the majority of the deviations along the x-axis was in the distal direction at 80% and 90%, respectively. Along the y-axis, the deviation was greater in group 1 in the oral direction (85%), in group 2 in the vestibular direction (60%). Along the z-axis, both groups deviated exclusively in the apical direction as shown in Fig. 7.

### Results of the questionnaires

To check the reliability of the questions, Cronbach's α was calculated for the individual subject areas. The values ranged from 0.747 to 0.947 in the areas design, assistance by the software, ease of use, planning outcome and future use. In the area of time and effort, Cronbach's α was 0.484.

**Fig. 8:**
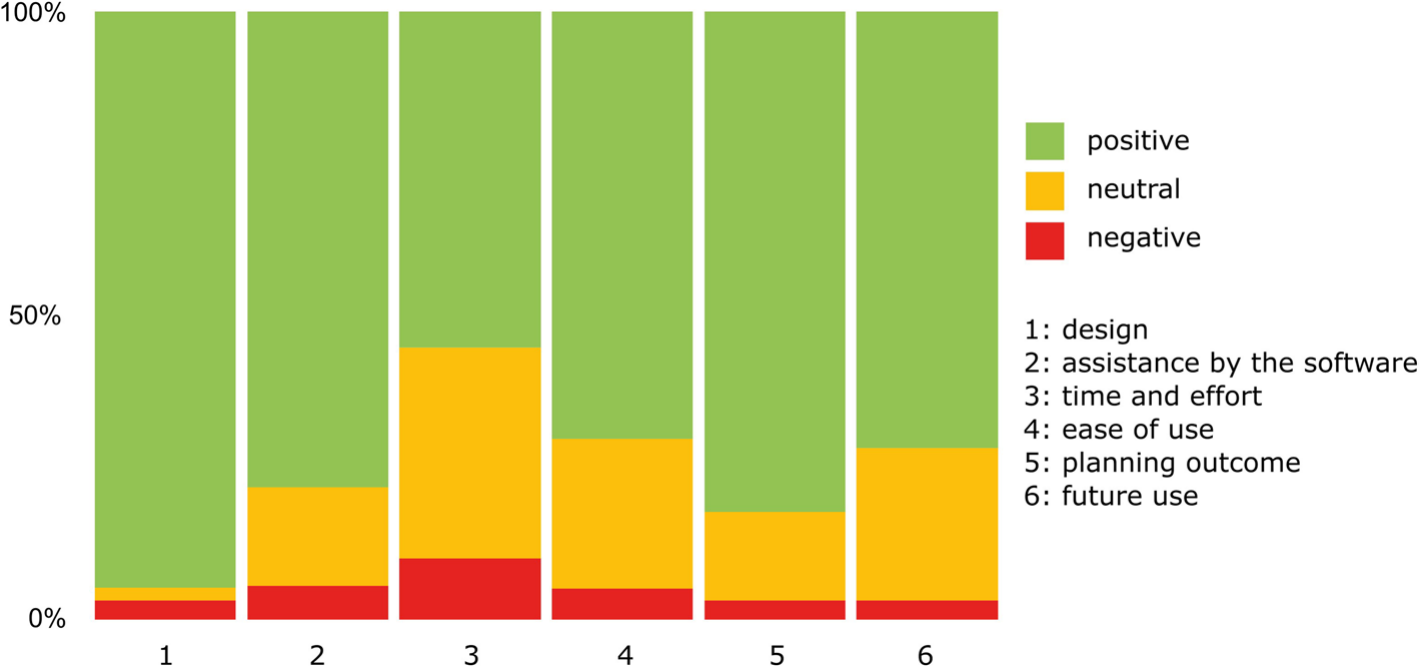
Results of the questionnaire about the software

The area design was rated best, with 95% positive responses. The area time and effort was the least positive, with only 55% positive responses (Fig. 8); the question "The software requires long calculation times between individual steps. (†)" was rated most negatively with a median of 5.00 in the entire questionnaire (Table 2).

**Table 2:** Evaluation of the individual questions of the CDX software; with Cronbach's α and median (Md); proportion of "negative", "positive" and "neutral" areas; recoded scale for comparison (†)

Subject area	α	Questions	Md	Proportion “positive” [%]	Proportion “neutral” [%]	Proportion “negative” [%]
Design	.947	The user interface appears clearly arranged	10.00	96.88	0.00	3.13
		The user interface has an appealing design	10.00	96.88	0.00	3.13
		The symbols of the action areas are fittingly designed	9.00	90.63	6.25	3.13
Assistance by the software	.902	The software guides me clearly through the planning process	9.00	90.63	6.25	3.13
		The software gives hints and explains certain functions	9.00	90.63	6.25	3.13
		The software reminds me of steps that still need to be done	8.00	75.00	21.88	3.13
		The software helps me to make decisions	8.00	59.38	34.38	6.25
		The software provides me with orienting parameters	8.00	81.25	9.38	6.25
		The software provides a warning of gross treatment errors (undercutting of minimum bone thicknesses and distances to anatomical structures, such as maxillary sinus/N. alveolaris inferior/adjacent teeth)	8.00	62.50	21.88	9.38
		The software suggests a suitable drilling template design	9.00	81.25	12.50	6.25
Time and effort	.484	The planning process is time-consuming. (†)	8.00	56.25	31.25	12.50
		The software is running stable	8.00	78.13	15.63	6.25
		The software requires long calculation times between individual steps. (†)	5.00	31.25	43.75	25.00
		The fenestration on the drill sleeve must be strongly adjusted. (†)	7.00	43.75	46.88	6.25
		Optimizing the drilling template is easy	8.00	65.63	34.38	0.00
Ease of use	.747	The general operation is self-explanatory	8.00	81.25	12.50	3.13
		I am confused/overwhelmed by the possibilities of the software. (†)	8.00	62.50	28.13	9.38
		The menu for selecting the correct implant is clearly laid out	8.00	68.75	28.13	3.13
		The distances between the implant and surrounding structures can be clearly read	8.00	50.00	43.75	3.13
		The software lets me correct previous steps in the process	9.00	84.38	9.38	6.25
Planning outcome	.933	The position of the implants in the virtual model can be traced through the visualization	9.00	81.25	15.63	3.13
		The result of the planning matches my expectations	8.50	81.25	15.63	3.13
		I am satisfied with my planning	9.00	84.38	12.50	3.13
Future use	.755	I intend to use the software again in the future	9.00	84.38	12.50	3.13
		Using the software strengthened/awakened my interest in implantology	9.00	84.38	12.50	3.13
		I trust myself to carry out correct planning by myself	7.00	46.88	50.00	3.13

The statistical analysis showed that the overall experience with the software was rated 74% positive, 21% neutral and 5% negative (Table 3).

**Table 3:** Evaluation of the subject areas of the CDX software; proportion of "negative", "positive" and "neutral" areas

Subject area	Proportion “positive” [%]	Proportion “neutral” [%]	Proportion “negative” [%]
Design	94.79	2.08	3.13
Assistance by the software	78.28	16.29	5.43
Time and effort	55.35	34.59	10.06
Ease of use	70.25	24.68	5.06
Planning outcome	82.29	14.58	3.13
Future use	71.88	25.00	3.13
All subject areas	73.97	20.58	5.45

## Discussion

The null hypothesis for the study is that there is no significant difference in the implant position between group 1 and 2.

In group 1, the mean deviation along the x-axis was -0.305 ± 0.373 mm, that along the y-axis was 0.433 ± 0.453 mm, that along the z-axis was -0.467 ± 0.310 mm, and for the total deviation was 0.893 ± 0.370 mm. A recent in vitro study compares the accuracy of FG implantation. The mean mesial-distal deviation at the implant shoulder was 0.160 ± 0.120 mm, the buccal-palatal deviation was 0.360 ± 0.220 mm, the apical-coronal deviation was 0.650 ± 0.310 mm, and the global coronal deviation was 0.770 ± 0.320 mm [18]. Thus, except for the z-axis, the deviations were slightly smaller than those in our study, but the difference was less than 0.200 mm for all values. Another study was able to measure mean deviations in the mesial-distal and the buccal-lingual direction. The mean deviation was 0.440 ± 0.780 mm in the distal direction and 0.230 ± 1.080 mm in the buccal direction for FG implantation in models [19]. The results also deviate only slightly from our measurements. However, the greater scattering of the values is notable here.

In group 2, the mean deviation along the x-axis was -0.381 ± 0.317 mm, that along the y-axis was -0.089 ± 0.879 mm, that along the z-axis was -0.643 ± 0.344 mm, and for the total deviation was 1.146 ± 0.456 mm. Guentsch et al. reported mean deviations at the level of the alveolar ridge of 0.140 ± 0.110 mm in the mesial-distal direction, 0.620 ± 0.150 mm in the buccal-lingual direction, and 0.200 ± 0.140 mm in the coronal-apical direction when HG implantation was performed in a model [20].

Compared with our work, these values were greater in the buccal-lingual direction but lower in the mesial-distal and coronal-apical directions. Differences of up to 0.531 mm were thus obtained.

Another study examined deviations in the mesial-distal and the buccal-lingual direction. For HG implantation in the models, the mean deviation was 0.330 ± 1.380 mm in the mesial direction and 0.620 ± 1.150 mm in the buccal direction [19]. In addition to the larger deviation in the bucco-lingual direction, the larger scatter of the values is also noticeable here.

Overall, the results of our study are comparable, with only minor differences. However, they cannot be fully compared with each other because of the different naming of the deviation directions [21].

According to the literature, to achieve a clinically acceptable result despite an unavoidable deviation of the implant position from the planned position, a safety distance of at least 2 mm from sensitive anatomical structures should be included [22]. It is difficult to achieve a deviation of less than 0.5 mm [23]. The deviations in our study were within the mentioned safety distance.

After comparing the groups, significant deviations in the implantation axes along the y- and z-axes and in the total deviation are noticeable. FG implantation is thus significantly more precise than HG implantation with initial marking of the implant position with the aid of the surgical template in the oro-vestibular direction and in terms of implantation depth. Thus, the null hypothesis can be rejected. The students tended to deviate from the reference position in the oro-vestibular direction as soon as implantation was performed without the surgical guide. Additionally, the students placed the implants deeper in the HG implantation than in the reference implantation. No significant difference was found regarding mesio-distal alignment. The fact that a greater degree of guided implant placement generally leads to greater precision has been demonstrated in several studies [11, 15, 16].

Regarding the results of the questionnaires, Cronbach´s α was in an acceptable range between 0.7 and 1 in the areas of design, assistance by the software, ease of use, planning outcome and future use [17, 24]. These values show that the individual questions can reliably determine their respective areas. In contrast, Cronbach's α for the domain of time and effort was below 0.5 (Table 2). Thus, these questions can identify this domain less reliably.

Although the design was rated highest overall in the survey, the difference between the individual domains in terms of negative ratings is small. Thus, the aspects of the software that are relevant for the clinical success of an implant placement, such as the planning result and the assistance, are perceived as equally suitable from the students' point of view.

The inclusion of implantology content in university teaching has resulted in students increasingly recognizing the importance of implant placement as a restoration in their later practice activities [25]. Due to the numerous offers of practical courses and various lecture series on implantology, it is not surprising that the students rated the topic of future use as positive (72%) and only once with a negative attitude per question. The majority of students can therefore imagine using the software again for future planning. The use of the software also strengthened or awakened the students' interest in implantology. A prior study, which included a survey of third-year students, revealed that the implementation of implant planning software in teaching is widely accepted [17].

In the field of implantology, various aspects should therefore be integrated into teaching at an early stage to be able to create a reliable basis for practical courses and further training after graduation [26].

The results of the survey indicate that students who encounter planning software for the first time and have only limited experience in implant planning are also able to operate it in a target-oriented manner within a short period of time.

Considering the methods applied in this study, it is noteworthy that some research uses a combination of different angles and distances to measure deviations [27]. In this study, deviations were determined using a 3D coordinate system with x, y and z axes [28].

Comparable studies have used resin models for implantation. Similarly, studies have been performed on human jaws [10, 23]. For our study, we decided to use duplicated resin models. Due to the manufacturing method, slight differences in the hardness of the individual models are conceivable.

Considering the study's limitations, our study investigated only FG implantation in comparison to HG implantation. In general, FH implantation is not suitable for beginners, especially in the aesthetic area. Implant placement in the anterior region represents the rehabilitation procedure with the highest requirements, as it requires consideration of not only functional and anatomical aspects but also aesthetic factors, which can present practitioners with challenging treatment scenarios [29]. Precise placement of an implant in the anterior region, taking into account prosthetic and aesthetic considerations, is crucial for achieving an aesthetically satisfactory outcome [29, 30]. Students and less experienced dentists can benefit from preoperative planning support in the precise realization of the intended implant position through FG and HG implantation techniques. In addition, several studies have already been published that addressed the differences between FG and FH implantations [15, 16, 31]. Nevertheless, exploring the potential impact of freehand implant placement on the outcomes of the study would be interesting.

Since a reference implantation was performed to determine the deviations, small deviations in the implant position are also conceivable here. Additionally, only one software was used to plan the implant position. Consequently, a comparison with other softwares for the same planning is not possible. Different implant planning software may result in data discrepancies due to differences in technology (data import, segmentation, data matching). The use of a single software program eliminated these errors. Nevertheless, a comparison with other software would be a useful addition in terms of teaching relevance. It's important to note that this aspect wasn't the study's focus, which centered on practical exercise outcomes.

Another limitation of this study is that the time spent by the participants on the individual steps of the implant procedure (including X-ray and intraoral scan matching, implant positioning planning, guide planning, fabrication, and implant placement) was not recorded. Studies have shown that for unexperienced practitioners, the average time required for implant placement ranges from 7.58 to 10.5 min [32, 33]. Another study reported that undergraduates spent a total of 57:34 min on the design and fabrication of the digital surgical guide [34].

In the future, it would therefore be useful to investigate the influence of the software on the accuracy of the planning and implementation by using different softwares. The planning and restoration of a larger gap situation, for example with a bridge construction, could also be used to investigate the influence of a lower orientation to the neighboring teeth on the implant positioning.

## Conclusions

Overall, the students achieved more accurate results with the full-guided implantation. In comparison, the students deviated in the vestibular direction along the y-axis, particularly with the half-guided implant placement, which meant that the intended oral positioning could not be maintained. In both groups, the implants were placed deeper along the z-axis than planned. Smaller deviations could be determined in the mesio-distal direction, since the neighboring teeth could additionally serve as orientation for positioning.

From the questionnaire it could be concluded that the software used is very user-friendly and therefore very suitable for teaching.

In summary, beginners should always favor full-guided implant placement, as long as it is clinically feasible.

## Supplementary Information

Supplementary Material 1

## Data Availability

All data generated, analysed and supporting the findings of this study are included within the paper. Upon explicit individual request, the authors will make the raw data sets used in the study available to interested parties.

## Abbreviations

3D: Three-dimensional
CBCT: Cone beam computed tomography
CDX: CoDiagnostiX (10.5, Straumann Group, Basel, Switzerland)
FG: Full-guided
FH: Freehand-guided
HG: Half-guided
IOS: Intraoral scan
