# Supplementary material for: Accuracy of full-guided versus half-guided implant procedures carried out with digital implant planning software by students as part of a university curriculum
Source: BMC Med Educ. 2024 Nov 15;24:1316. doi: 10.1186/s12909-024-06280-7 (PMC11566595; doi:10.1186/s12909-024-06280-7)
Supplement: Supplementary file 1 — Supplementary Material 1 [file 12909_2024_6280_MOESM1_ESM.pdf]

Universitätsklinikum Erlangen

Questionnaire on implantation software

Please answer all of the following questions. Please select only one answer per question.

	Strongly disagree / not applicable	1	2	3	4	5	6	7	8	9	10	Strongly agree / applicable
		○	○	○	○	○	○	○	○	○	○	
**Design**												
• The user interface appears clearly arranged.		○	○	○	○	○	○	○	○	○	○	
• The user interface has an appealing design.		○	○	○	○	○	○	○	○	○	○	
• The symbols of the action areas are fittingly designed.		○	○	○	○	○	○	○	○	○	○	
**Assistance by the software**												
• The software guides me clearly through the planning process.		○	○	○	○	○	○	○	○	○	○	
• The software gives hints and explains certain functions.		○	○	○	○	○	○	○	○	○	○	
• The software reminds me of steps that still need to be done.		○	○	○	○	○	○	○	○	○	○	
• The software helps me to make decisions.		○	○	○	○	○	○	○	○	○	○	
• The software provides me with orienting parameters.		○	○	○	○	○	○	○	○	○	○	
• The software provides a warning of gross treatment errors (undercutting of minimum bone thicknesses and distances to anatomical structures, such as maxillary sinus/N. alveolaris inferior/adjacent teeth).		○	○	○	○	○	○	○	○	○	○	
• The software suggests a suitable drilling template design.		○	○	○	○	○	○	○	○	○	○	
**Time and effort**												
• The planning process is time-consuming.		○	○	○	○	○	○	○	○	○	○	
• The software is running stable.		○	○	○	○	○	○	○	○	○	○	
• The software requires long calculation times between individual steps.		○	○	○	○	○	○	○	○	○	○	
• The fenestration on the drill sleeve must be strongly adjusted.		○	○	○	○	○	○	○	○	○	○	
• Optimizing the drilling template is easy.		○	○	○	○	○	○	○	○	○	○	
**Ease of use**												
• The general operation is self-explanatory.		○	○	○	○	○	○	○	○	○	○	
• I am confused/overwhelmed by the possibilities of the software.		○	○	○	○	○	○	○	○	○	○	
• The menu for selecting the correct implant is clearly laid out.		○	○	○	○	○	○	○	○	○	○	
• The distances between the implant and surrounding structures can be clearly read.		○	○	○	○	○	○	○	○	○	○	
• The software lets me correct previous steps in the process.		○	○	○	○	○	○	○	○	○	○	
**Planning outcome**												
• The position of the implants in the virtual model can be traced through the visualization.		○	○	○	○	○	○	○	○	○	○	
• The result of the planning matches my expectations.		○	○	○	○	○	○	○	○	○	○	
• I am satisfied with my planning.		○	○	○	○	○	○	○	○	○	○	
**Future use**												
• I intend to use the software again in the future.		○	○	○	○	○	○	○	○	○	○	
• Using the software strengthened/awakened my interest in implantology.		○	○	○	○	○	○	○	○	○	○	

- I trust myself to undertake accurate planning independently. ○ ○ ○ ○ ○ ○ ○ ○ ○ ○
